# Treatment outcomes in palliative care: the TOPCare study. A mixed methods phase III randomised controlled trial to assess the effectiveness of a nurse-led palliative care intervention for HIV positive patients on antiretroviral therapy

**DOI:** 10.1186/1471-2334-12-288

**Published:** 2012-11-06

**Authors:** Keira Lowther, Victoria Simms, Lucy Selman, Lorraine Sherr, Liz Gwyther, Hellen Kariuki, Aabid Ahmed, Zipporah Ali, Rachel Jenkins, Irene J Higginson, Richard Harding

**Affiliations:** 1 Cicely Saunders Institute, Department of Palliative Care and Rehabilitation, Kings College London, London, UK; 2 London School of Hygiene and Tropical Medicine, London, UK; 3 University College London, London, UK; 4 Department of Family Medicine, University of Cape Town, Cape Town, South Africa; 5 Department of Medical Physiology, University of Nairobi, Nairobi, Kenya; 6 Bomu Medical Centre, Mombasa, Kenya; 7 Kenyan Hospice Palliative Care Association, Nairobi, Kenya; 8 Institute of Psychiatry, Kings College London, London, UK

**Keywords:** HIV, ART, Palliative care, Africa, Evaluation

## Abstract

**Background:**

Patients with HIV/AIDS on Antiretroviral Therapy (ART) suffer from physical, psychological and spiritual problems. Despite international policy explicitly stating that a multidimensional approach such as palliative care should be delivered throughout the disease trajectory and alongside treatment, the effectiveness of this approach has not been tested in ART-experienced populations.

**Methods/design:**

This mixed methods study uses a Randomised Controlled Trial (RCT) to test the null hypothesis that receipt of palliative care in addition to standard HIV care does not affect pain compared to standard care alone. An additional qualitative component will explore the mechanism of action and participant experience. The sample size is designed to detect a statistically significant decrease in reported pain, determined by a two tailed test and a p value of ≤0.05. Recruited patients will be adults on ART for more than one month, who report significant pain or symptoms which have lasted for more than two weeks (as measured by the African Palliative Care Association (APCA) African Palliative Outcome Scale (POS)). The intervention under trial is palliative care delivered by an existing HIV facility nurse trained to a set standard. Following an initial pilot the study will be delivered in two African countries, using two parallel independent Phase III clinical RCTs. Qualitative data will be collected from semi structured interviews and documentation from clinical encounters, to explore the experience of receiving palliative care in this context.

**Discussion:**

The data provided by this study will provide evidence to inform the improvement of outcomes for people living with HIV and on ART in Africa.

ClinicalTrials.gov Identifier: NCT01608802

## Background

In 2009, the global number of people with HIV was 33.3 million (95% CI 31.4 million- 35.3 million) with the majority in sub Saharan Africa, where 22.5 million adults and children live with HIV infection [1]. Improved treatment and care programs are urgently required [2]. Despite recent treatment guideline changes, which have raised the recommended CD4 threshold for initiation of antiretroviral therapy (ART) [3] (indicating that the population of ART patients should be expected to increase), the World Health Organization (WHO) reports that the number of eligible patients receiving treatment has increased from 28% in 2008 to 36% in 2009 worldwide. Under the previous treatment guidelines, this proportion would have been 52% in 2009 [4]. This increase in the number of eligible patients will become a mounting clinical challenge.

Studies in Brazil [5], Malawi [6], USA [7], UK [8] and South Africa [9] demonstrate that people living with HIV continue to experience a significant symptom burden after ART initiation. This was clearly demonstrated by a large UK study of HIV outpatients [10], who reported physical, psychological and global distress and symptom counts which remained unaffected by treatment status, suggesting that accessing effective treatment for the virus might not be sufficient to alleviate suffering. The prevalence of symptoms and associated distress in this patient group can be caused by toxicities from the ART itself [11-13], symptoms of opportunistic infections and co-morbidities or HIV disease [7,10,14]. People with HIV report lower quality of life [6,15-17], attributed to their physical and psychological symptom burden. In Australia, patients with HIV receiving ART had a significantly higher prevalence of depression when compared with HIV negative patients recruited from the same clinics [18]. Data from a UK study confirm this, with psychological distress reported by 75% of a sample of HIV outpatients, regardless of treatment status [8]. In Sweden, although physical health status improved after ART initiation, patients reported a deterioration in emotional quality of life, associated with an increase in the number of adverse reactions they experienced [19]. In Tanzania 53% of patients attending a clinic (of whom the majority were receiving ART), had palliative care needs [20].

Palliative care is defined by the WHO [21] as an essential component of care for people living with HIV. Palliative care includes the assessment and treatment of pain and other symptoms, whether physical, psychosocial or spiritual in nature, delivered alongside treatment. Leading experts have highlighted the false dichotomy of palliative care treatment versus cure, particularly among the vulnerable poor in developing countries [22-27]. The WHO clearly identifies an urgent need for holistic palliative care integrated with treatment for those suffering from chronic diseases such as HIV. This has been reiterated by UNAIDS, which has identified the misconception that palliative care is only appropriate for patients at the end of life, and is therefore working towards the availability of palliative care for all people living with HIV [28].

The WHO public health model of palliative care, developed in 1990, integrates palliative care into all of society, from community level to expert palliative care provision and in existing healthcare structures [29]. However, the pioneering service provision attempts and advocacy for this model are hampered by a lack of experimental research, and a lack of standardised measures and protocols, which would facilitate a more robust approach to healthcare evaluation and delivery [30]. For successful implementation, the WHO public health model of palliative care needs robust evidence of effectiveness in relevant contexts.

There are wider reasons beyond patient quality of life that indicate the importance of attention to palliative care-related problems. Depression and treatment of side effects are associated with non-adherence to ART [31,32], which increases viral resistance, rebound and infectiousness [33]. A recent systematic review of palliative care-related problems at HIV diagnosis identified significant physical and psychological symptoms among newly diagnosed HIV positive patients [34]. A systematic review of the evidence for effectiveness of HIV palliative care found that it improves anxiety, pain, symptoms, and insight but that the evidence was generated almost exclusively in the pre-ART era and in high income countries [24]. There are currently no known trials of palliative care for patients with HIV on ART. This lack of evidence originating from low and middle-income countries is problematic in light of the great disease burden in these areas.

We therefore aim to inform HIV service provision by conducting a randomised controlled trial (RCT) to assess the effectiveness of palliative care for HIV outpatients on ART, and present here the protocol for the phase III RCT with a qualitative component.

## Methods/design

### Aim and objectives

#### Aim

We aim to evaluate the efficacy, in terms of reported pain of a nurse led palliative care intervention for HIV patients on ART. Nurses will receive two weeks in depth training in palliative care and support and supervision from an experienced palliative care mentor. Two trials are being conducted, one in Mombasa, a low-income setting, and one in Cape Town, a middle-income setting. In line with guidance from the Medical Research Council (MRC) on the evaluation of complex interventions, qualitative data will also be collected to complement the quantitative data and address the question of how the intervention might work [35].

#### Objectives

1. To investigate whether self-report pain and symptoms significantly improve for HIV positive patients under palliative care compared to those in standard HIV care.

2. To compare self-report adherence to ART under palliative care compared to standard HIV care.

3. To compare self-report health-related quality of life under palliative care compared to standard HIV care.

4. To compare additional multidimensional palliative care outcomes (psychological, social and spiritual wellbeing) under palliative care compared to standard HIV care.

5. To understand the process of receiving palliative care and identify any specific component which may be the most effective aspect (i.e. access to strong analgesia, multidimensional assessment, access to multidisciplinary team).

### Study design

The study will consist of two fully powered, independent phase III clinical RCT’s preceded by a pilot. Each trial will be powered and conducted in parallel to a common research design protocol, thus providing evidence of outcomes in two different settings.

Patients will be randomly allocated to standard HIV care or standard HIV care plus palliative care. The palliative care will be delivered within the existing HIV clinic, and by existing staff, using an integrated model, with the option to refer to a specialist palliative care provider for complex cases. The study has been designed with measures to minimise potential contamination. Once the study nurses have been trained in palliative care, they will only see the patients allocated to the intervention, and will not be required to work in the main clinic until completion of the trial.

The design will be longitudinal, using repeated measures. Patient-centred outcomes will be measured using quantitative questionnaires.

#### Outcomes

The primary outcome is self-reported pain after four months. The secondary outcomes are health-related quality of life; adherence to treatment; risk behaviours; and psychological morbidity and the core domains of palliative care as defined by the WHO (physical, psychological, social and spiritual well-being) as measured by the APCA African POS.

Patients will be followed up for four months and most outcomes are measured at baseline and monthly intervals, described below.

#### Control

Patients randomly allocated to the control arm will receive the usual clinical care delivered by the HIV clinic. Nurses who have had no exposure to palliative care will provide this service.

In Kenya this consists of six-monthly clinical assessments once ART has been established, with investigations and treatment for any relevant symptoms or problems. Patient may attend the clinic for medications refill only or may request more frequent appointments if they experience a problem. In South Africa, patients attend the public hospital monthly for a brief appointment to refill medications. They may present for additional appointments as necessary.

#### Intervention

Patients randomly allocated to the intervention arm will receive clinical care from a nurse who has received two weeks’ training in palliative care and ongoing clinical support and supervision from experienced palliative care providers. The palliative care trained nurse will also have the option to refer complex cases for management at a hospice.

### Minimum package of palliative care

Patients allocated to the intervention arm will receive an initial clinical assessment , followed by either one further visit or phone call within the first week, one further visit or phone call within the second week and one visit per month thereafter. The focus of the care provided will be on holistic assessment and management of physical, psychological, spiritual and social problems. This minimum package of care can be supplemented by additional clinical support as required on a case-by-case basis.

### Intervention nurse training

Training will be provided by local expert palliative care sites, and will be delivered in an intensive 2-week period, tailored to the needs of patients living with HIV who are not necessarily in advanced stages and who are currently on ART.

The training will be designed using national and international guidelines [36,37] to ensure that the nurses are prepared to address the specific palliative care dimensions and needs of those living with HIV and taking ART, with specific focus on the management of common HIV symptoms. Within these two weeks, the nurses will also gain clinical experience, working in a palliative care setting.

### Standardised assessment form

Assessment and management of multidimensional problems is a central part of palliative care. The trained nurse will use a standardised assessment form, developed by the study team drawing on tools and models used in sub Saharan Africa, to assess the domains of palliative care as defined by the WHO [21] and monitor adherence.

### Clinical support

The nurse will receive weekly clinical support from an experienced palliative care provider, where all cases will be reviewed and decisions appraised. Drugs needed for the intervention group not stocked in the study site pharmacy will be dispensed by the supporting palliative care service.

### Referral

As part of clinical support, referral to the hospice will be available for patients in the intervention arm whose needs are “complex” or apparently refractory and who require assessment or intervention from the palliative care specialist partner site. Criteria for referral will be established prior to the study launch, and recorded in the clinical documentation.

#### Sample size

We used the largest known dataset generated by the APCA African POS (detailed below) to derive expected levels of change used in the sample size calculation. The dataset consisted of baseline data from a longitudinal evaluation of care and support which recruited 1337 HIV patients in Uganda and Kenya [38]. Stata v10.0 was used for calculations.

Sample size was first calculated based on change in the pain item of the APCA African POS, as the primary outcome. A clinically significant change in an APCA African POS item is a change of 1 point so this is identified as the expected change [39]. We propose a sample size of 56 per arm to be able to demonstrate a difference between treatment conditions for pain, symptoms, and both physical and psychological dimensions of quality of life (the latter as a mediating variable in a model of adherence). The study will recruit 60 patients per arm (120 total in each country), allowing for 6% drop-out/attrition [40].

#### Settings

The two participating HIV care facilities (one in Kenya and one in South Africa) are highly experienced HIV and ART service providers, with proven longevity. The study site in Kenya is a private clinic funded by private donations in a deprived area of Mombasa. Regular appointments are made to see a nurse for a health check and ART refill, with frequency dependant on adherence, opportunistic infections and CD4. Patients have access to a physician if referred by the triaging nurse, and good access to essential medicines. The South African study site is a government-run clinic in district township, south-east of Cape Town. Patients have monthly appointments for a health check and ART refill, and have similar access to clinic physicians as in Kenya – upon nurse referral. Access to essential medicines is mostly reliable, but is hampered by economic constraints necessitating short prescriptions. In both countries, the providers of the palliative care training and support are longstanding local experts. In Kenya, training was provided by Kenyan Hospice and Palliative Care Association (KEHPCA) with support from an expert from Coast Hospice in Mombasa. In South Africa, training was provided by Hospice Palliative Care Association (HPCA) and support was provided by an expert from Helderberg Hospice.

#### Recruitment and consent

##### Screening and recruitment

Because of organisational and logistical differences between the two study sites, recruitment will be performed in a slightly different way, whilst maintaining the integrity of random sampling and respecting the logistical constraints, patient flow and unique pressures of each clinic.

In Kenya, the initial patient to be screened will be chosen randomly using the number allocated to them at registration that day, and a number selected from a random number table. Once the initial patient has been screened, patients will be screened consecutively. In South Africa patients are simply screened consecutively according to the order in which they present at the clinic.

### Inclusion/exclusion criteria

In both countries, patients included in the study must be adult patients (i.e. aged 18 years or above), with known HIV infection who have been receiving ART for more than one month.

Patients meeting these criteria will be screened by a researcher, using first two items of the APCA African POS. If they report a score of 3, 4 or 5 on either (possible 0–5 score), they will be asked whether they have been experiencing the pain or symptoms for 2 weeks or longer. Patients who answer in the affirmative will be eligible for participation. This is in order to identify those with symptoms which suggest a chronic rather than acute problem. The researcher will then outline the study content and demands and ask whether they would like to consent to participate. This process will take place every day during the data collection period, although the time of the day will vary randomly in order to collect a representative sample of the clinic population.

In Kenya, potential participants will be screened for eligibility from a list of all patients currently in the clinic, in the triage room of the comprehensive care unit, where all HIV positive patients are seen. In South Africa, all patients in the clinic are HIV positive and on ART, therefore the researcher will sit in the clinic room with the nurse and will screen each potential participant as they attend their appointment. All patients who are called to see the nurse will be screened consecutively and invited to consent if eligible.

In summary, to be eligible for participation, patients must have sufficient cognitive ability to answer the outcome tool questions using either verbal or hand responses (see data collection below), be receiving ART for at least one month (based on clinic records) and must report either pain or symptoms of 3, 4 or 5 on the APCA African POS (i.e. severe to overwhelming) for longer than two weeks. Exclusion criteria include pain and symptoms with a duration of less than 2 weeks, receiving ART for Prevention of Mother To Child Transmission (PMTCT) of HIV, or for some other reason not related to personal clinical need or if the patient does not speak either English or Kiswahili in Kenya, and English, isiXhosa or Afrikaans in South Africa.

### Consent

If eligible, the patient is taken through the information and consent sheet in a separate private space, respecting the patient flow of the clinic and the demands on patient time. Patients will have an information sheet read aloud to them, and will then be asked to sign or mark a consent form. All information and consent forms will be translated into the principal local languages. Consent using either a signature or mark will be obtained according to local custom or patient preference. All consent forms will be kept securely at the facility, and stored separately from outcome data. Each patient will be allocated a unique identifying number, kept by the researcher.

All participants will have the right to withdraw, without notice, at any point. Participants who withdraw from the intervention arm will continue to receive the intervention for the full four months. They will be asked whether they want their data to be withdrawn. If so, it will be deleted from the database and the paper copy will be destroyed. After quantitative data collection has finished, the dataset will be anonymised and from that point it will no longer be possible to identify individuals and withdraw data.

A record of the number of patients not agreeing to participate will be kept at each facility in accordance with CONSORT guidelines for the reporting of RCTs [41].

The sample for the qualitative interviews will be purposively drawn from intervention (n=20) and control patients (n=10) based on the clinical and demographic patient characteristics and results of the quantitative data collection i.e. psychological well-being. This will attempt to ensure a representative sample in terms of demographic and clinical data, and also in terms of psychological well-being and response to the intervention.

### Randomisation

Consenting patients will be administered the initial assessment and subsequently randomised to intervention or control group. This is done using block randomisation to ensure a manageable work load for the nurse delivering the intervention, with 40 per block. Each study site has been issued with three sealable pots containing 20 pieces of paper with “I” for intervention and 20 pieces of paper with “C” for control. Once a piece of paper has been blindly selected by the researcher, the patients is informed whether they are in the control or intervention group and this is recorded on a form which is kept separately from the data collection tools and records. The piece of paper is discarded and the process continues until the pot is finished, when the next pot is then used. The allocation to control or intervention is not blinded as it would be impossible to maintain this blinding when the intervention was delivered.

### Compensation

Study participants are not given financial compensation for their time, but they are given $5USD towards transport expenses for data collection appointments. Transport for intervention patients clinical appointments (of which there are at least two more than research appointments) are not reimbursed as this may influence the outcome. For comfort, participants are given a drink and small snack on arrival for their data collection appointments.

### Ethical considerations

Ethical approval has been sought and secured from Kings College London Research Ethics Committee (BDM/10/11-31), University of Cape Town (019/2011), Ministry of Health of the Western Cape (Research request ID: 10252) and Kenyan Medical Research Institute (KEMRI/RES/7/3/1).

**Figure 1:**
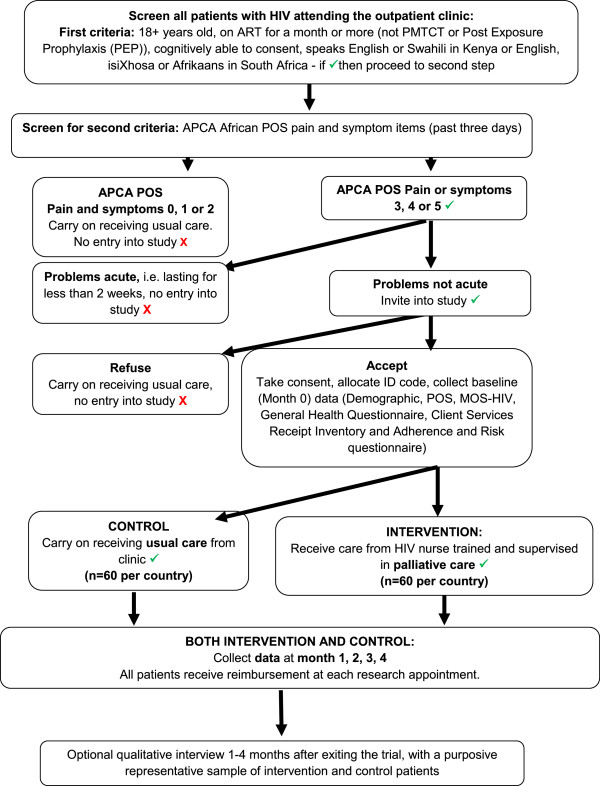
Study flow chart.

A distress protocol will be used, with participants being offered the opportunity to cease the interview if they become distressed during questioning. They will be able to decide to restart or abandon the interview at their own discretion. All information gathered during data collection will be confidential, except in the situation of a participant or someone related to the participant being at risk, in which case the information will be acted upon. Please see Figure 1 for study flow chart.

### Data collection and analysis

Data will be collected by local researchers following the study protocol. They will be trained in quantitative and qualitative data collection methods, and will receive specific training for each tool used in this study.

### Tools

The data collection tools are:

#### Patient demographic record

##### Administered once only, at month 0

This brief record will be used to record age, gender, couple status, number of children, number of financial dependents, education, recent CD4, date of ART initiation, WHO staging data and whether the patient is receiving TB treatment. This questionnaire also includes assessment of relative socioeconomic status, as used in the Demographic Health Surveys [42].

#### APCA AFRICAN POS (10 items)+ eastern Co-operative global performance scale (ECOG) (1 item)

##### Administered at months 0, 1, 2, 3 and 4

The original Palliative Outcome Scale (POS) was developed at King’s College London [43,44] (further information and resources are available at http://pos-pal.org/). The APCA African POS was culturally adapted from the original POS during development in ten centres in six sub-Saharan African countries [45]. The ten items of the APCA African POS address the primary physical, emotional and spiritual concerns of patients and families with progressive disease using a scoring method appropriate for a range of literacy skills. Each item is scored on a scale of 0–5, and can be scored verbally or using the “hand” method commonly used in Africa. Using this method, a closed fist represents “0”, moving up to an open hand scoring “5”. These methods have been validated among 300 patents under palliative care in Africa with a correlation of around 0.9 for verbal vs hand scoring methods for both pain scores and symptom scores. For those with no family carer, family items are scored as “0”, i.e. no problem [45]. The tool is sensitive to change and acceptable to patients, family and staff. Its brief nature makes it highly appropriate for use in a large-scale longitudinal study [46].

The ECOG is a single item measure of performance and is the most widely used performance measure [47]. Scores range from 0 (normal activity) to 4 (unable to get out of bed). It has been used in previous work amongst HIV positive people in Africa [48].

#### Medical outcome study -HIV (MOS-HIV quality of life measure)

##### Administered at months 0, 1, 2, 3 and 4

The MOS-HIV was originally developed as a general health questionnaire in the USA [49]. A modified HIV-specific version was developed and widely used. It has been culturally adapted to the Ugandan HIV setting [50] and has been used in Rwandan [51] Zimbabwean [52] and Ugandan populations [53]. The 35 items address the domains of role function, pain, physical functioning, cognitive functioning, social functioning, general health perception, mental health, health distress and vitality.

#### General health questionnaire-12 (GHQ)

##### Administered at months 0, 1, 2, 3 and 4

The GHQ-12 is a widely used measure of psychological morbidity [54]. It is the shortest validated version of the original GHQ, which was a 60-item instrument when it was first designed in the 1970s [55]. The GHQ-12 has been translated and validated into many languages including Kenyan Kiswahili [56].

#### Adherence and risk questionnaire

##### Administered at months 0, 2 and 4 only

The adherence and risk questionnaire has been used for research in the UK, predominately with gay men [10,57]. The risk section of the questionnaire consists of three questions detailing sexual partners in the past four months, detailing unprotected sex and unprotected sex with people of unknown HIV status. The adherence questions asks participants how many ART doses they have missed in the past week.

#### Client services receipt inventory (CSRI)

##### Administered at months 0, 1, 2, 3 and 4

The CSRI aims to explore the components of care received by patients. The version of the CSRI used in this study is based was adapted from versions used in other palliative care research and adapted for use in Africa [58,59]. The version used for this study has been simplified in that instead of documenting time, the researcher records whether the client has received a service or not as a binary outcome.

#### Qualitative interview schedule

##### Administered for selected patients at month 4

The interview schedule was developed in collaboration with international researchers experienced in HIV and palliative care research and local researchers with an in depth knowledge of the delivery of palliative care at the study sites. It is designed to explore the intervention and control patients’ perspectives on participating in a randomised trial, the intervention patients’ experiences of receiving palliative care, and to determine the potential mechanism of action of the intervention.

### Piloting

All tools were professionally translated into local languages and checked for consistency and accuracy by local researchers in each country and piloted with 35 patients in Kenya. This checked for clarity of questions, time required for data collection, patient burden and availability of requested information.

### Quantitative data collection

Data collection will be conducted in the usual place of care, i.e. at home or at the clinic. For all tools, data will be collected as close as possible to the monthly timetable, with a two week grace period either side. Data collection appointments will be co-ordinated with clinical appointments where possible for minimal patient disruption.

### Qualitative data collection

The qualitative data collection will be conducted between one and four months after quantitative data collection finishes, to minimise recall bias. Interviews will be digitally recorded and translated and transcribed by experienced local experts. Each transcription will be checked and validated by the researcher who conducted the interview.

### Data management and quality assurance

A researcher will be employed at each participating centre. This person will be responsible for managing recruitment, data collection and data entry. Fortnightly study team conference calls will be used to ensure that all queries are promptly resolved, attended by all members. All quantitative data will be double-entered into purpose designed EpiData databases. Discrepancies will be resolved by referring back to the original questionnaires.

Following data checking and cleaning, the data will be imported into Stata for analysis at KCL. Qualitative transcripts will be imported into NVivo (version 9) for analysis. All hard data will be stored for at least seven years in accordance with the UK Data Protection Act 1998.

### Analysis

#### Quantitative analysis

Data will be described in two stages:

1) Cross sectional baseline analysis: descriptive analysis of demographic and clinical variables, baseline primary and secondary outcomes for the two groups (standard care and palliative care). Continuous variables will be presented as mean (standard deviation), median (range); categorical variables will be described as proportions. Baseline scores for primary and secondary outcome variables for two groups will be compared using two-sample t-test (or Mann–Whitney U test if non-normal distribution) for continuous data and Chi square test for categorical data.

2) Summary measures: change in primary and secondary outcomes from baseline to the final time point will be summarized, using paired t-tests or the non-parametric equivalent.

Longitudinal analysis will be performed to address the primary study objectives of comparing pain and other symptoms, (objective 1) and subsequently adherence (objective 2), health related quality of life (objective 3) and multidimensional palliative care outcomes such as psychological and spiritual well-being (objective 4), in the intervention and control groups. Factors of the APCA African POS were identified from APCA POS data from similar samples (high prevalence of HIV) in Sub Saharan Africa [60]. Three factors were identified which covered 1) physical and psychological symptoms, 2) interpersonal dimension and 3) the existential dimension. They will be analysed for differences between intervention and control groups. For continuous outcomes, the change in scores for two groups will be compared using general linear model with adjustment of baseline differences and covariates (demographics and clinical variables). For categorical outcomes, the two-group comparisons will be implemented using generalized linear model and adjusting for design effects, the repeated measures will be taken into account using generalized estimating equations (GEE) technique.

The analysis will follow the principle of intention-to-treat. The p-values reported will be two-tailed and an alpha level of 0.05 will be used to assess statistical significance. The analysis will be carried out using non-missing data. The pattern of missing data and drop-outs will be investigated and sensitivity analysis will be performed to assess their impacts on outcomes. If the impact is great, a missing data strategy will be put in place using mean horizontal imputation and last value carried forward.

Further descriptive analysis will be conducted to determine the common features and differences between the three palliative care facilities, and the services received as recorded in the CSRI. Analysis of the CSRI will also allow us to determine a dosage effect for receipt of palliative care, as well as allow an evaluation of the palliative care received with respect to outcomes.

#### Qualitative analysis

Analysis of translated transcripts will be conducted using thematic content analysis [61]. The coding frame will be developed initially deductively, using themes from the topic guide, combined with inductive themes which emerge from the data, which will be generated iteratively as the analysis progresses. The coding will be conducted separately by two researchers, one from the study site and one from the UK, and then merged, to ensure a comprehensive and culturally sensitive framework.

The preliminary findings will be cross checked by the researcher based at the study site and presented to the study team in each country for proxy member checking to improve validity [62].

A model of the mechanism of the psychosocial aspect of palliative care will be constructed on the basis of the data if possible and relevant.

### Dissemination

The audience for dissemination will include collaborating centres, participants, HIV clinicians, academic teaching staff in Africa, local community groups and non-governmental organisations, academic global research audiences, Ministries of Health and international non-governmental organisations.

## Discussion

The data provided by this study will provide evidence to inform the provision of palliative care for people living with HIV and on ART, building on our previous work describing the palliative care problems of this group [8,10,24,46].

Aspects of this study are unique and will contribute to the body of knowledge. The choice of study design, the first RCT in this field, will provide a clear answer to the question of whether palliative care for this group is effective and relevant. The multi-country aspect will illuminate the differences between low and middle income countries, and Southern and Eastern Africa, without inviting direct comparison of outcomes of two very different countries. Researchers in both countries will be locally recruited and trained by staff from King’s College London, building local capacity. Steps have been taken to reduce potential bias, including using locally validated tools, which have been translated in all three study languages by experienced translators. Researchers have been trained on aspects of data collection, bias minimisation and potential sources of contamination and data will be double entered to avoid inaccuracies from human error.

The publication of this protocol presents increased transparency in the aims and objectives of the study and analysis of the generated data.

## Abbreviations

APCA: African palliative care association; ART: Anti retroviral therapy; CSRI: Client services receipt inventory; ECOG: Eastern co-operative global performance scale; GHQ: General health questionnaire; HIV: Human immunodeficiency virus; HPCA: Hospice and palliative care association; KEHPCA: Kenyan hospice and palliative care association; MOS-HIV: Medical outcomes study HIV; MRC: Medical research council; PEP: Post exposure prophylaxis; PMTCT: Prevention of mother to child transmission; POS: Palliative outcomes scale; RCT: Randomised controlled trial; TB: Tuberculosis; WHO: World health organisation.

## Competing interests

The authors declared that they have no competing interest.

## Authors’ contributions

RH and VS conceived of the original study with input from LS, LG, HK, AA, ZA, RJ and IJH. KL, LS, RH and VS drafted the manuscript with contributions from, LG, HK, AA, ZA, RJ and IJH. All authors read and approved the final manuscript.

## Pre-publication history

The pre-publication history for this paper can be accessed here:

http://www.biomedcentral.com/1471-2334/12/288/prepub
